# Supplementary material for: Overweight and obesity among women at reproductive age in Cambodia: Data analysis of Cambodia Demographic and Health Survey 2014
Source: PLOS Glob Public Health. 2023 Mar 31;3(3):e0001768. doi: 10.1371/journal.pgph.0001768 (PMC10065241; doi:10.1371/journal.pgph.0001768)
Supplement: Attachment — Submitted filename: Response to reviewers.docx [file pgph.0001768.s001.docx]

**Authors’ point-by-point responses to the editor and reviewers**

**PGPH-D-22-01749**
Overweight and Obesity among Women at Reproductive Age 15-49 Years Old in Cambodia: Data Analysis of Cambodia Demographic and Health Survey 2014
PLOS Global Public Health

1- Please review your reference list to ensure that it is complete and correct. If you have cited papers that have been retracted, please include the rationale for doing so in the manuscript text, or remove these references and replace them with relevant current references. Any changes to the reference list should be mentioned in the rebuttal letter that accompanies your revised manuscript. If you need to cite a retracted article, indicate the article’s retracted status in the References list and also include a citation and full reference for the retraction notice

Thanks. We have provided the following citation [page 5, line 105, line 121, line 128, and page 6 line 139] to support our references and highlighted in the revised manuscript:

- **Reference #5**: National Institute of Statistics. Directorate General for HC. International ICF. Cambodia Demographic and Health Survey 2014. Phnom Penh, Cambodia: National Institute of Statistics/Cambodia, Directorate General for Health/Cambodia, and ICF International; 2015. 2014
- **Reference #14**: DHS Program. The Demographic and Health Surveys (DHS) Program; ICF: Rockville, MD, USA, 2020; Accessed on 28 March, 2022 at <https://dhsprogram.com/data/available-datasets.cfm>
- **Reference #15:** BOWLER P: CUNNINGHAM, A. and JARDINE, N.(editors). Romanticism and the sciences. Cambridge University Press, Cambridge: 1990. Pp xxii, 345; illustrated. Price:£ 15, US $19.95. ISBN: 0-521-35685-7 (paperback). In.; 1992.
- **Reference #16**: World Health Organization. Physical status: The use of and interpretation of anthropometry, Report of a WHO Expert Committee. WHO, 1995

2- Please insert an Ethics Statement at the beginning of your Methods section, under a subheading 'Ethics Statement'. It must include:
a) (For human participants/donors) - A statement that formal consent was obtained (must state whether verbal/written) OR the reason consent was not obtained (e.g. anonymity). NOTE: If child participants, the statement must declare that formal consent was obtained from the parent/guardian.

Thanks. We have moved the ethical statement to the beginning of the Methods section as suggested under the Subheading ‘**Ethics Statemen**t’ [page 5, lines 103-11] as follows. Also, revision has been made to reflect the journal requirement as highlighted in the revised manuscript.

3- We have noticed that you have uploaded Supporting Information files, but you have not included a list of legends. Please add a full list of legends for your Supporting Information files after the references list.

Thank you. We did correct a mistake.

**Reviewers' Comments to the Authors:**

**Reviewer 1**

The greatest strength of the study is that it used a nationally representative sample that made the findings of study valid for Cambodian population. Although the findings of the study is similar to the findings of other international studies of the same population, the result can be used as a base line value for comparison in future.

Some minor revision should be considered. Please see the comment below:

1. Please add reference/s to Methods section for the sampling procedure if there is any published methods for CDHS 2014 survey.

Thanks for the suggestion. We have addressed this above as suggested and followed the Journal Requirement [page 5, line 121].

- **Reference #5**: National Institute of Statistics. Directorate General for HC. International ICF. Cambodia Demographic and Health Survey 2014. Phnom Penh, Cambodia: National Institute of Statistics/Cambodia, Directorate General for Health/Cambodia, and ICF International; 2015. 2014

2. Outcome and explanatory variables were defined well in the paper but how the data was collected has not been mentioned explicitly. It was mentioned under the ‘Data Source’ paragraph that participants were interviewed. However, how the body weight and height was measures was not mention in the paper. Please provide details or referred to any published method that was used for the anthropometric measurement.

We have addressed this as suggested and highlighted in the revised manuscript about women’s anthropometric measurement in the Subheading ‘**Data Source and Procedures’** [page 6, lines 124-128].

3. Statistical Analysis and Result sections were explicit.

Thank you.

4. Grammatical issues:

Dr. Jonathan A. Muir has revised and made corrections grammatically and accordingly as suggested.

a) First line of Abstract- you may consider rewriting the sentence as follows “Overweight and obesity are known the risk factors for….”

Thanks. We have addressed this as suggested and highlighted in the revised manuscript

b) Please consider rewriting the third sentence of the 1st paragraph of the Abstract.

Revised as suggested.

c) In the 2nd sentence of 2nd paragraph of the Abstract- not sure what authors wanted mean by the sentence. Please consider rewriting the sentence.

Thanks. We have revised the sentences as suggested.

d) 2nd sentence of 1st paragraph of page 4- Author may consider rewriting the sentence as below- “Overweight and obese women of reproductive age (WRA) group (15-49 years) are at increased risk for ……”

Thanks. Revision has been made and highlighted

**Reviewer 2**

1. Suggestion revising the manuscript title ‘Overweight and Obesity among Women at Reproductive Age 15-49 Years Old in Cambodia: Data Analysis of Cambodia Demographic and Health Survey 2014’

We agree with the suggestion and it has been revised accordingly.

2. Suggestion first line of the introduction can be placed later or should omit from the first sentence ‘The World Health Organization (WHO) defined overweight and obesity as abnormal body mass index (BMI), calculated as an individual weight in kilograms divided by the square of height in meters (kg/m2)’?

Thanks. We have removed it as suggested

3. This process is just a replication from the main survey. So, can you please add the references here?

Thanks. We have provided a source of citation in the revised manuscript.

4. How the household wealth indexing was carried out by CDHS 2014 survey?

We have added in Methods in the revised manuscript. “*The household wealth index was calculated based on household assets and dwelling characteristics using Principal Component Analysis (PCA), and the scores were given into five categories of wealth quintiles (poorest, poorer, medium, richer, and richest) each comprising 20% of the population. Then, the original variable was then recombined into three categories including richer/richest, middle, and poorest/poorer.”*

5. Can you tell it is simply Non-smoker? or simply, can it be a smoker?

Thanks. We have agreed with the suggestion and it has been revised in the manuscript in Table 1.

6. Did you mean the Barrier method by the term “Traditional method”? Please clarify

The traditional methods mean periodic abstinence and withdrawal
